# Precision Immunotherapy Utilizing Adapter CAR-T Cells (AdCAR-T) in Metastatic Breast Cancer Leads to Target Specific Lysis

**DOI:** 10.3390/cancers16010168

**Published:** 2023-12-29

**Authors:** Cansu E. Önder, Moustafa Moustafa-Oglou, Sarah M. Schröder, Andreas D. Hartkopf, André Koch, Christian M. Seitz

**Affiliations:** 1 Research Institute for Women’s Health, University of Tübingen, 72076 Tübingen, Germany; cansu.onder@live.de; 2 Department of Pediatric Oncology and Hematology, University Hospital Tübingen, 72076 Tübingen, Germany; moustafa.moustafa-oglou@med.uni-tuebingen.de; 3 Department of Otorhinolaryngology, Head and Neck Surgery, University of Ulm, 89081 Ulm, Germany; 4 Department of Peptide-Based Immunotherapy, University and University Hospital Tübingen, 72076 Tübingen, Germany; 5 Department of Women’s Health, University of Tübingen, 72076 Tübingen, Germany; 6 Cluster of Excellence iFIT (EXC 2180) “Image-Guided and Functionally Instructed Tumor Therapies”, University of Tübingen, 72076 Tübingen, Germany; 7 German Cancer Consortium (DKTK), Partner Site Tübingen, a Partnership between German Cancer Research Center (DKFZ) and University Hospital Tübingen, 81675 Munich, Germany

**Keywords:** precision immunotherapy, adapter CAR-T cells, metastasis, breast cancer, organoid culture, pleural effusion, cancer biology, patient-derived organoids

## Abstract

**Simple Summary:**

The development of pleural effusion is a common debilitating occurrence during metastasized breast cancer. Malignant cells in pleural effusions originating from the primary tumor suggest the spread of the disease and can serve as a model for metastatic breast cancer. Hence, we established three-dimensional organoid lines from four patients with malignant pleural effusion. Patient-derived organoids were characterized by flow cytometry for individual target antigen expression profiles. Adapter CAR-T cells (AdCAR-T) and biotinylated monoclonal antibodies were evaluated to specifically target patient-derived organoids and assess responses in a personalized fashion. This study demonstrates the feasibility of precision immunotherapy utilizing AdCAR-T to target patient-individualized antigen patterns.

**Abstract:**

A frequent symptom of metastasized breast cancer (BC) includes the development of malignant pleural effusion (MPE), which contains malignant cells derived from the primary tumor site. The poor prognosis of MPE in metastasized BC indicates the necessity for dependable precision oncology and the importance of models representing the heterogenous nature of metastatic BC. In this study, we cultured MPE-derived metastatic tumor cells from four advanced BC patients using organoid technology. We assessed the expression of tumor-associated antigens on MPE-derived organoid lines by flow cytometry (FC). Based on an individual antigen expression pattern, patient-derived organoids were treated with adapter CAR-T cells (AdCAR-T) and biotinylated monoclonal antibodies targeting CD276, HER2, EGFR, TROP2, or EpCAM. Co-culture assays revealed specific organoid lysis by AdCAR-T depending on individual antigen expression patterns. Our results demonstrate that MPE-derived organoids can serve as a reliable tool for assessing the efficacy of AdCAR-T on metastatic BC in a patient-individualized manner. This approach could potentially be applied in a preclinical setting to instruct therapy decisions. Further, our study demonstrates the feasibility of precision immunotherapy utilizing AdCAR-T to target patient-individualized antigen patterns.

## 1. Introduction

Globally, breast cancer (BC) stands out as the most diagnosed cancer and a leading contributor to cancer-related fatalities in women [1]. BC presents itself with four molecular subtypes (luminal A, luminal B, HER2-enriched, and basal-like) based on a 50-gene expression signature (PAM50) [2]. Besides this molecular classification, a more traditional classification based on the immunohistochemistry expression of key proteins such as estrogen receptor (ER), progesterone receptor (PR), human epidermal growth factor receptor 2 (HER2), and the proliferation marker Ki67, are commonly employed (reviewed in [3]). BC cells can acquire invasiveness, leading to the formation of metastases, as they spread through the bloodstream or lymphatic system to various organs and sites in the body. Primary BC has been extensively studied and generally has a positive prognosis. In contrast, metastatic BC presents numerous challenges, including the presence of diverse cell types and resistance to treatment. These factors often contribute to the failure of therapeutic interventions [4].

Therapy decisions usually depend on the features of primary tumors, as metastatic biopsies are relatively rare. Yet, primary tumors may not fully represent the heterogeneous characteristics of metastatic tumors. Frequently, these attributes demonstrate inconsistency in phenotypic markers [5]. Consequently, therapy recommendations based on the characteristics of primary tumors alone might lead to poorer outcomes [6,7].

Malignant pleural effusion (MPE), which describes the presence of malignant cells in the pleural cavity, develops in 7% of BC patients [8,9]. The quality of life and prognosis of patients suffering from MPE are relatively poor and defined by chest pain and breathing difficulties [10]. However, as MPE contains metastatic BC cells and can be obtained through a simple puncture, it offers more options for metastatic biopsies [11]. Moreover, malignant cells in MPEs can serve as a source for the establishment of in vitro models that represent the characteristics of metastasized BC to improve therapy outcomes and approach precision oncology. The most common approaches include cell lines and patient-derived xenograft (PDX) models. While cell lines are widely used in BC research but are not capable of predicting drug response in patients, PDX models are invaluable for translational research but are expensive and limited in efficiency [12]. 

Recently, patient-derived BC organoids have been shown to be valuable three-dimensional models for research and personalized oncology, as they represent the characteristics of their respective origin and can be used for long-term culturing [13,14]. Cultured in an extracellular matrix, organoids of different sources have already been used in various methods, such as high-throughput drug assays [13,15].

In the area of BC treatment, standard therapeutic approaches involve surgery, chemotherapy, radiotherapy, endocrine therapy, and targeted therapy [16]. The latter approach includes immunotherapy, which has risen as a crucial element, presenting promising effectiveness and minimal safety concerns [17]. The emergence of genetically modified T cell therapies, incorporating a chimeric antigen receptor (CAR) has achieved remarkable success in long-lasting clinical responses among individuals with hematologic malignancies [18]. This sparked immense enthusiasm in the potential to address various types of cancer including BC. CAR-T cell therapy, a type of immunotherapy derived from adoptive T cell transfer, has been established to harness the patient’s own immune cells to fight cancer by triggering antigen-specific cytotoxic response [19].

Conventional CAR constructs are limited in their ability to provide adjustable cytotoxicity and flexible selectivity against heterogenous tumors. These limitations result in potential risks such as uncontrolled CAR-T expansion, depletion of normal tissues expressing the target antigen, CAR-T exhaustion, and lack of activation toward antigen-negative tumor cells [20,21,22].

One sophisticated approach to circumvent these limitations is to separate target antigen recognition from CAR-T activation through the introduction of adapter molecules (AMs) and adapter CARs (AdCARs) [23]. These AMs combine the functional components of an antigen-binding moiety and a CAR-binding moiety. Being able to conjugate clinically approved therapeutical antibodies, effectively turning them into AMs, enables flexible redirection of CAR-T cells toward different tumor-associated antigens (TAAs) and furthermore introduces a controllable on/off-switch.

In previous studies, we have demonstrated the remarkable effectiveness of AdCAR-T cells in vitro and in vivo by specifically targeting tumors associated with a broad spectrum of tumor-associated antigens (TAAs) across different cancer types [23,24]. This CAR technology provides the advantage of universal targeting of various cancer types and furthermore allows for the finely adjustable modulation of effector function and ultimately addresses the challenge of immune evasion due to antigen loss.

While CAR-T cell therapy has proven great achievements in hematologic malignancies, its effectiveness in battling solid tumors, including BC, still has room for improvement. In a previous study, we cultured tumor cells derived from MPE of advanced BC patients employing organoid technology and generated metastatic BC patient-derived organoids (MBC-PDOs). MBC-PDOs were then used as a platform to perform drug screenings applying various inhibitors [14]. 

In this study, we cultured MBC-PDOs and analyzed their antigen expression patterns utilizing flow cytometry (FC). Our goal was to utilize MBC-PDOs as a screening platform to demonstrate feasibility of an AdCAR-T-based precision immunotherapy approach for flexible targeting of various tumor-associated target antigens. In the future, research on patient-derived models could have enormous potential for clinical applications, such as extending patients’ survival time and improving their quality of life.

## 2. Materials and Methods

### 2.1. Patient Cohort

All MBC-PDOs were previously established and characterized [14]. The study was approved by the Ethics Committee of the Eberhard Karl University of Tübingen (ethical approval 288/2022BO2) and is compliant with all relevant ethical regulations regarding research involving human participants. For full patient characteristics see Table S1.

### 2.2. Culturing 2D Cell Lines

BC cell lines MDA-MB-468 and MCF-7 were acquired from American Type Culture Collection (Manassas, VA, USA, HTB-22 and HTB-132). Cells were handled in DMEM-FBS (Dulbecco’s Modified Eagle Medium (41965-062), containing 10% FBS (10270-106), 1% Pen/Strep (15140-122) from Thermo Fisher Scientific, Waltham, MA, USA). Cells were recurrently checked for mycoplasma using a PCR Detection Kit (abm, Richmond, BC, Canada, G238).

### 2.3. Freezing and Thawing of CAR-T Cells

Cultured cells were centrifuged at 350× *g* for 5 min and resuspended in human serum albumin (HSA, CSL Behring GmbH, Marburg, Germany, 4356500002) containing 10% dimethyl sulfoxide (DMSO, PanReac Applichem, Darmstadt, Germany, A3672) at a cell density of 1–10 × 10^7^ cells/mL; 1 mL aliquots were frozen overnight at −80 °C and subsequently transferred to liquid nitrogen storage. To thaw cells, frozen aliquots were rapidly thawed in a 37 °C water bath, diluted in pre-warmed TexMACS™ medium (Miltenyi Biotec, Bergisch Gladbach, Germany, 31870) without interleukins or antibiotics and centrifuged at 350× *g* for 5 min. Then, T cells were resuspended in TexMACS™ medium without supplementation at a density of 4 × 10^6^ cells/mL and incubated for 4 h before adding IL-7/IL-15-supplemented (Miltenyi Biotec, Bergisch Gladbach, Germany, 130-095-367 and 130-093-955) TexMACS^TM^ media to achieve a cell density of 1 × 10^6^ cells/mL.

### 2.4. Isolation and Transduction of T Cells

Peripheral blood mononuclear cells (PBMCs) were isolated from freshly collected peripheral blood samples via Ficoll centrifugation (Biocoll^®^, BIO&SELL GmbH, Nürnberg, Germany, L 6115) from healthy voluntary donors at the University Children’s Hospital Tübingen. T cell isolation was performed using anti-CD4 and anti-CD8 microbeads (Miltenyi Biotec, Bergisch Gladbach, Germany, 130-045-101 and 130-045-201), subsequently activated with TransAct^TM^ (Miltenyi Biotec, Bergisch Gladbach, Germany, 130-128-758) and cultivated in TexMACS^TM^ media supplemented with 10 ng/mL IL-7 and 5 ng/mL IL-15. After 36 h, activated T cells were transduced with AdCAR (LLE-CAR) lentivirus (provided by Miltenyi Biotec, Bergisch Gladbach, Germany) at a multiplicity of infection (MOI) of 3. Transduced T cells were maintained at 0.5–2 × 10^6^ cells/mL in IL-7/IL-15-supplemented TexMACS^TM^ media and furthermore monitored for lactate. CAR transduction efficiency was determined by flow cytometry at day 7 using AdCAR detection reagent (provided by Miltenyi Biotec, Bergisch Gladbach, Germany).

### 2.5. Adapter Molecule Conjugation

Biotin adapter conjugation was achieved at 30 °C for 1 h in DPBS buffer (Thermo Fisher Scientific, Waltham, MA, USA, 14190-094) using 5-fold molar excess of biotin-LC-LC-NHS (Thermo Fisher Scientific, Waltham, MA, USA, CAS-No. 89889-52-1). This was followed by separation of the antibody/label mix on a Sephadex G25 column (Cytiva Europe GmbH, Freiburg, Germany, 17085101). Protein holding fractions were collected, analyzed by absorption at 280 nm, and subsequently united. Successful conjugation was confirmed via flow cytometry on cell lines expressing the target.

### 2.6. Organoid Culture Setup

Cryopreserved MBC-PDOs from our previous study were used for the study [14]. For the setup of organoid cultures, the required amount of cell suspension was mixed with basement membrane extract (BME; Cultrex Reduced Growth Factor Basement Membrane Extract, Type 2 Select, Bio-techne, Minneapolis, MN, USA, 3533-005-02) at a ratio of 30% cell suspension to 70% BME; 20 µL droplets were seeded on 48-well plates and placed upside down in an incubator (37 °C, 5% CO_2_) to solidify for 30 min. BC culture medium (BCM; Table S2, composition previously described [13,25]) was added to each well and renewed every 3–4 days. Cells were incubated at 37 °C and 5% CO_2_ and pictures were taken frequently with EVOS M7000 microscope (Thermo Fisher Scientific, Waltham, MA, USA).

### 2.7. Passaging of MBC-PDOs

MBC-PDOs were passaged every 7 to 21 days, depend on organoid size and density. Organoids were recovered from the wells by resuspending the BME droplets in ice-cold DPBS containing 5 µM Y-27632 (DPBS/Y-27632). The organoid suspension was centrifuged at 500× *g* for 10 min and the supernatant was discarded. The BME-organoid pellet was dispersed with 1 mL of TrypLE™ Express Enzyme (1X; Thermo Fisher Scientific, Waltham, MA, USA, 12604013) at 37 °C in a water bath for 5 min. The suspension was then centrifuged (500× *g* for 10 min) and the supernatant was discarded. For further culture, the desired amount of cell pellet was resuspended in AdvDMEM+++ and mixed with BME at a ratio of 30% cell suspension to 70% BME and cultured as described above. To stock organoids, passaged cells were cryopreserved in Recovery™ Cell Culture Freezing Medium (Thermo Fisher Scientific, Waltham, MA, USA, 12648010) and stored in cryovials in liquid nitrogen.

### 2.8. Generation of Lentiviral Vector

Luciferase and GFP containing lentivirus were produced as previously described [23]. After lipofection (Lipofectamine 3000, Thermo Fisher) of Lenti-XTM 293 T (Clontech/ TaKaRa Bio Company, San Jose, CA, USA, 631231) with second-generation packaging plasmid, VSV-G envelope plasmid and transfer plasmid, lentivirus containing supernatants were concentrated (Lenti-X concentrator, TaKaRa Bio Company, San Jose, CA, USA, 631231) and cryopreserved at −80 °C.

### 2.9. Viral Transduction and Sorting of MBC-PDOs

Third-generation-based lentiviral vector transfer plasmids containing luciferase and GFP were kindly provided by Irmela Jeremias, Helmholtz Center Munich, Munich, Germany (Irmela Jeremias, Helmholtz Center Munich, Germany, 12260). Lentiviral particles were produced as described above. All MBC-PDO lines were transduced at an MOI of 3. Subsequent transgene expression was analyzed by flow cytometry using the co-expressed fluorescent protein. Transduced cells were enriched by bulk fluorescence-activated cell sorting (FACS).

### 2.10. Flow Cytometry

Flow cytometry analysis was performed by staining 0.2 × 10^6^ cells in fluorescence- activated cell sorting (FACS) tubes. The following antibodies by Miltenyi Biotec (Bergisch Gladbach, Germany) were used to determine antigen expression. Anti-CD47 (anti-human, Biotin, 130-101-342), anti-CD66 (anti-human, Biotin, 130-093-156), anti-CD112 (anti-human, Biotin, 130-109-000), anti-CD133 (anti-human, Biotin, 130-112-193), anti-CD146 (anti-human, Biotin, 130-092-850), anti-CD171 (anti-human, Biotin, 130-100-702), anti-CD276 (anti-human, Biotin, 130-118-579), anti-ROR1 (anti-human, Biotin, 130-118-018), anti-TROP2 (anti-human, Biotin, 130-115-096), anti-CD326 (EpCAM) (anti-human, Biotin, 130-111-114), HER2 (biotin-conjugated Trastuzumab, Kanjinti, 1144554A), and EGFR (biotin conjugated Cetuximab, Erbitux, G0157D) (Table S3). CliniMACS^®^ buffer (Miltenyi Biotec, Bergisch Gladbach, Germany, 700-25) containing antibodies at an equimolar concentration of 20 µg/mL was added to each sample. The cells were furthermore stained with anti-Biotin (APC; Miltenyi Biotec, Bergisch Gladbach, Germany, 130-111-069) and analyzed via BD FACSCanto II (BD Biosciences, Franklin Lakes, NJ, USA).

### 2.11. Two-Dimensional Luciferase-Based Cytotoxicity Assay (LCA)

Tumor cells were plated in complete RPMI media (Thermo Fisher Scientific, Waltham, MA, USA, 31870) in white 96-well flat-bottomed plates (Greiner Holding, Kremsmünster, Austria, 655083) with 15,000 cells per well and were co-cultured with AdCAR-T cells at a 2:1 ratio. At timepoint 0 h, adapter molecules and synthetic D-luciferin (Sigma Aldrich, St. Louis, MO, USA, L9504) were added to each well at a final concentration of 10 ng/mL and 4 µg/mL, respectively. Bioluminescence was assessed after 24 h, 48 h, and 72 h using the Tecan SPARK microplate reader (Perkin Elmer, Waltham, MA, USA, 30086376) at 37 °C. Lysis was evaluated by the relative luminescence to tumor control wells without AdCAR-T cells.

### 2.12. Real-Time Impedance-Based Cytotoxicity Assay (ICA)

The impedance-based Real-Time Cytotoxicity Analyzer (RTCA) xCELLigence device (ACEA Biosciences Inc., Santa Clara, CA, USA, 106-0534) was used to assess label-free real-time cytotoxicity [23]. MCF-7 and MD-MBA-468 cell lines were plated at 30,000 cells per well in RPMI 1640-based complete media in 96-well electronic microtiter plates E-Plate^®^ 96 (ACEA Biosciences Inc., Santa Clara, CA, USA, 2801035). After 24 h, effector cells were added according to indicated effector to target ratio. Therapeutic antibodies (AMs) were used at 10 ng/mL. Plates were incubated under 37 °C, 95% humidity, and 5% CO_2_, and impedance was assessed every 15 min for 24 h.

### 2.13. Three-Dimensional Luciferase-Based Cytotoxicity Assay (LCA)

For the AdCAR-T cell treatment of MBC-PDOs and MCF-7, cells were cultured 7 to 21 days as described in Section 2.7. The day before treatment, MBC-PDOs were recovered from the BME droplets by incubating the droplets in 1 mg/mL Dispase (Sigma-Aldrich, St. Louis, MO, USA, D4693) at 37 °C for 20 min. Droplet suspensions were diluted in 1% BSA and centrifuged at 250× *g* for 10 min. The supernatant was removed, and organoid pellets were resuspended in the required amount of assay medium (BCM without Nicotinamide and Y-27632). Per well, 125 µL of organoid suspensions was seeded in 96-well plates (clear plates, 353072; white plates, 136102; both from Thermo Fisher Scientific, Waltham, MA, USA) coated with 40 µL BME-advDMEM (in a ratio of 50% BME and 50% AdvDMEM+++).

CAR-T cells were thawed the day before treatment and cultured in TexMacs^TM^ medium lacking cytokines and including 10% FBS at 37 °C for 4 h. Cells were then diluted to a concentration of 1 × 10^6^ cells/mL with TexMacs^TM^ supplemented with IL-7 (10 ng/mL), IL-15 (5 ng/mL), penicillin (100 units/mL)/streptomycin (100 µg/mL), and cultured at 37 °C until the following day.

The following day, MBC-PDO cells were counted by the addition of TrypLE to one well per line. Organoids dispersed into single cells at 37 °C for 10 min and were pelleted and resuspended in 100 µL of AdvDMEM+++. MBC-PDO and CAR-T cells were counted using the Bio-Rad TC20™ Automated Cell Counter (Bio-Rad Laboratories, Inc., Hercules, CA, USA) according to manufacturer’s protocol.

Diluted in assay medium, 25 µL of CAR-T cells (various E:T ratios), 25 µL of biotinylated antibodies (80 ng/mL), and 25 µL D-Luciferin (2 ng/µL) were added to the wells, reaching a total volume of 200 µL. Wells of clear plates received assay medium instead of D-Luciferin; also, control wells received assay medium as a replacement for CAR-T cells and/or biotinylated antibodies. Readouts were performed after 24 h, 48 h, and 72 h by measuring the luminescence (white plates) with a Varioskan LUX (Thermo Fisher Scientific, Waltham, MA, USA) and capturing brightfield and fluorescence images (clear plates) of MCF-7 organoids and MBC-PDOs. Assays were carried out in multiple technical replicates, and results were normalized to untreated controls.

## 3. Results

### 3.1. Design of AdCAR-T Cell System and Functional Targeting of BC Cell Lines

**Figure 1:**
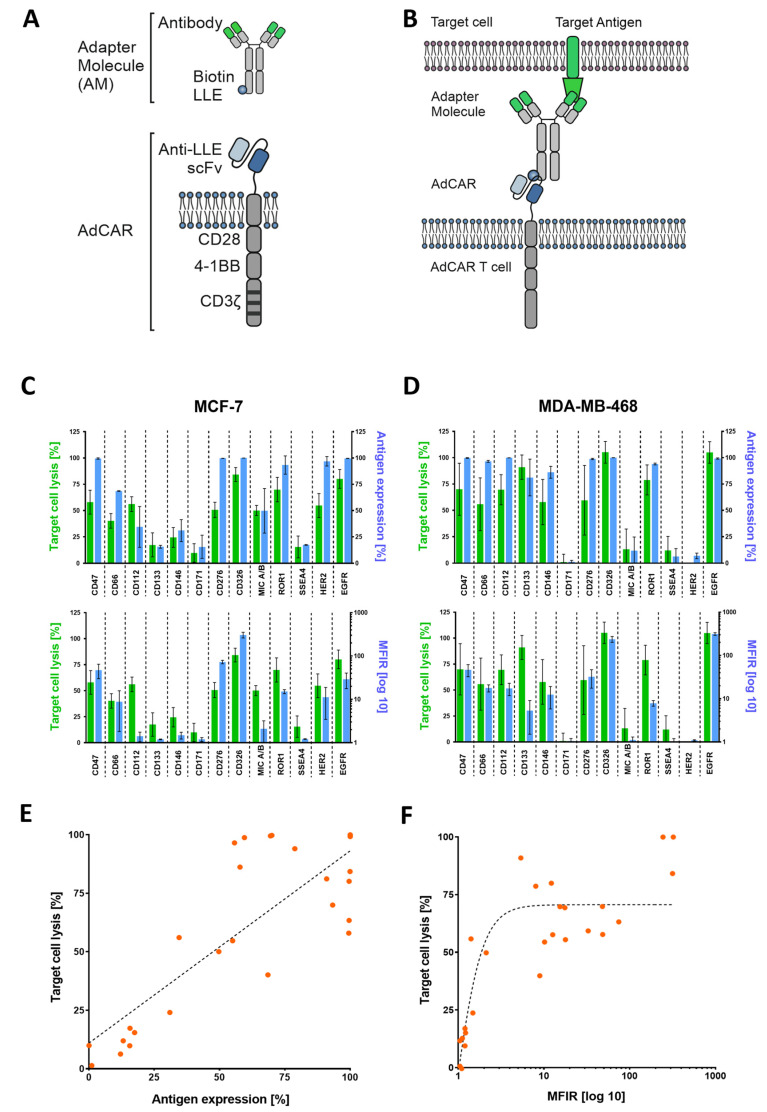
AdCAR-T cell design and functional targeting of BC cell lines using adapter molecules (AMs). (**A**) Schematic illustration of an adapter molecule and AdCAR-T cell receptor. (**B**) AdCAR-T cells are directed towards the target antigen via LLE-conjugated biotinylated antibodies referred to as AMs. (**C**,**D**) Comparison of target cell lysis of MCF-7 and MB-MDA-468 (green bars) to either overtone positive antigen expression (upper blue bars) or mean fluorescence intensity ratio (MFIR) log 10 of antigen expression (lower blue bars). Target cell lysis was determined via luciferin-based cytotoxicity assay after 48 h with an E:T ratio of 2:1. Antigen screening with respective antibodies was performed via flow cytometry (FC) and is represented by mean values (*n* = 6) ± SD. (**E**) Correlation between target cell lysis and antigen expression of both cell lines analyzed via linear regression resulted in a Spearman correlation coefficient of 0.66. (**F**) Analyzing exponential regression for the correlation of target cell lysis and MFIR log 10 of both cell lines showed a Spearman coefficient of 0.77. All data used in (**E**,**F**) are depicted by mean values (*n* = 6).

The AdCAR-T system was designed by conjugating the murine clone mBio3-derived scFvs on a third-generation CAR backbone consisting of extracellular spacer domains, CD28 and 4-1BB co-stimulatory, as well as the CD3-ζ signaling domain (Figure 1A) [23]. Unlike conventional CAR-T cells, the AdCAR-T cell system is designed to separate target antigen recognition from T cell activation. This can be achieved by a two-component approach, where AdCAR-T cells identify the linker–labeled–epitope (LLE) of biotin-conjugated adapter molecules (AMs), effectively utilizing their specific antigen-binding capacity (Figure 1B). This two-component system enables targeting a multitude of antigens expressed on target cells, utilizing biotin-conjugated monoclonal antibodies (mAbs) that are in clinical use (Table S3).

To validate the effectiveness of AdCAR-T cells against a range of target antigens, we conducted a cytotoxicity assay using luciferase-expressing MCF-7 (Figure 1C) and MD-MBA-468 (Figure 1D) cell lines. Expression levels of potential BC target antigens such as HER2, EGFR, and TROP2, among others, were assessed via flow cytometry (FC). Immunophenotyping (FC) was performed using the very same biotinylated mAbs, that were subsequently used in cytotoxicity assays. Target cell lysis (of MCF-7 and MDA-MB-468) was compared to the target antigen expression, which reveals a clear correlation between high antigen expression and target cell lysis (Figure 1E,F). When plotting target cell lysis against antigen expression using linear and exponential regression models, the Spearman correlation coefficient value indicates 0.66 and 0.77, respectively. To further validate our observation, we replicated these findings in an impedance-based cytotoxicity assay (ICA), as Supplementary Figure S1 shows.

### 3.2. Cultivation and Characterization of MBC-PDOs Expressing Luciferase and GFP

**Figure 2:**
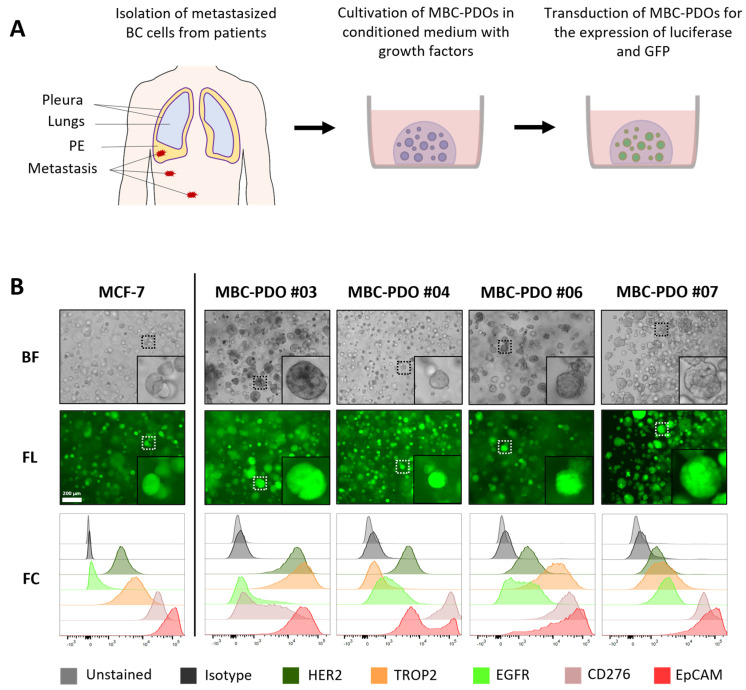
Cultivation and characterization of metastatic breast cancer (BC) patient-derived organoids (MBC-PDO), originated from malignant pleural effusion (MPE). (**A**) Schematic overview of isolation of metastatic BC cells from MPEs, as well as cultivation in BME droplets and viral transduction of MBC-PDOs. (**B**) Brightfield (BF) and fluorescence (FL) images as well as FC analysis of luciferase- and GFP-expressing MCF-7 and MBC-PDO #03, #04, #06, and #07. Scale bar: 200 µm.

In order to test the efficacy of AdCAR-T cell lysis on BC patient-derived cells, we used our previously established in vitro models for metastasized BC derived from pleural effusions [14]. Figure 2A shows a schematic overview of the isolation of MBC cells from pleural effusions and the establishment of MBC-PDO cultures. For better visualization and analysis of cell lysis, MBC-PDO lines were virally transduced to express luciferase and GFP (Figure 2A,B). The transduction efficiency of each generated MBC-PDO line was visualized via brightfield and fluorescence microscopy (Figure 2B).

To assess the antigen patterns of MBC-PDOs, we immune-profiled all transduced and sorted MBC-PDOs. Immunophenotyping (FC) was performed using biotinylated mAbs that were subsequently used in cytotoxicity assays (Figure 2B). The target antigen panel was chosen based on the expression levels found in BC and their suitability for CAR-T cell therapy. We focused on the following tumor-related antigens found in BC: HER2, TROP2, EGFR, CD276, and EpCAM. Figure 2B shows the expression of the surface antigens in normalized histograms, in which MCF-7 organoids served as a control.

Based on histological characteristics and receptor status (ERα, PR, and HER2), BC is categorized into different subtypes [3]. The receptor status of BC determines the therapy the patients receive and indicates the prognosis. While receptor-positive tumors can be treated with endocrine therapy, or targeted with antibodies and inhibitors, receptor-negative (triple-negative) BC has a poorer prognosis as it is treated with chemotherapy only.

Here, all MBC-PDO lines presented low to medium levels of HER2, with MBC-PDO #03 having the highest level of HER2. The signal for EpCAM was the highest in all five samples (lower panel, red curves), while the other antigens were represented in different quantities. Consequently, the MBC-PDO lines may serve as well-suited in vitro models for the application of personalized AdCAR treatment of metastatic BC cells.

### 3.3. Implementation of AdCAR Treatment of Organoids

**Figure 3:**
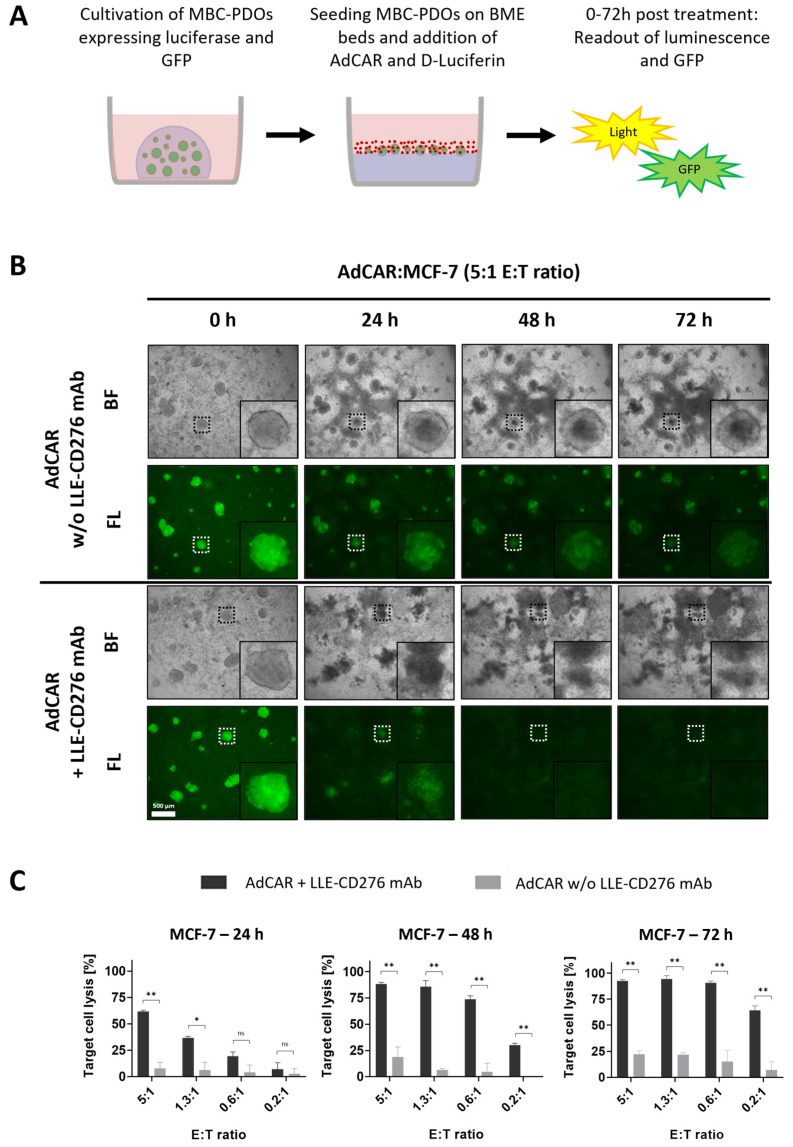
AdCAR treatment of MCF-7 organoids expressing luciferase and GFP. (**A**) Schematic overview of the experimental setup. MCF-7 and MBC-PDOs (green) expressing luciferase and GFP were seeded on BME beds and treated with AdCAR-T cells (red) and corresponding LLE-CD276 mAb. Readouts were performed on luminescence and GFP after 24 h, 48 h, and 72 h using a plate reader and a fluorescence microscope. (**B**) Brightfield (BF) and fluorescence (FL) images of GFP-expressing MCF-7 organoids treated with AdCAR-T cells without (−) and with (+) LLE-CD276 mAb. Scale bar: 500 µm. (**C**) Target cell lysis of MCF-7 organoids treated with AdCAR-T cells with (black bars) and without (gray bars) LLE-CD276 mAb over 72 h. Target cell lysis efficiency was determined by luciferase activity of remaining cells. Data shown represent the mean ± SD of biological triplicates (*n* = 3). Negative values were set to 0. Statistical analysis was performed using paired *t*-test. ns, not significant. * = *p* ≤ 0.05; ** = *p* ≤ 0.01.

We explored the potential of MBC-PDOs as in vitro models of BC metastasis and their suitability to evaluate AdCAR-T-mediated cytotoxicity. To demonstrate the universal antigen-specific effector function of AdCAR-T cells in vitro, we used adapter molecules in the LLE-mAb format targeted to various tumor-associated antigens expressed by the malignant cells. To implement the application of AdCAR treatment, we first tested various E:T ratios on GFP- and luciferase-expressing MCF-7 organoids. As previously shown, MCF-7 cells strongly express the target antigen CD276 (Figure 2B). Hence, organoids were grown, harvested, and seeded on BME beds to be treated with AdCAR-T cells and LLE-CD276 mAb (Figure 3A). AdCAR-T cells were incubated at different E:T ratios (ranging from 5:1 to 0.2:1). The LLE-mAb concentration was used at 10 ng/mL in all experiments. 

Figure 3B presents brightfield and fluorescence images of MCF-7 organoids treated with AdCAR with and without LLE-CD276 mAb. GFP signal and luciferase activity of viable cells were captured after 24 h, 48 h, and 72 h (Figure 3B,C). In the absence of LLE-CD276 mAb, organoids stayed intact even at the highest E:T ratio of 5:1 (Figure 3B), hence, there was no unspecific lysis detected. Yet, the GFP signal decreased slightly, as the organoids were covered by proliferated AdCAR-T cells. In the presence of LLE-CD276 mAb however, MCF-7 organoids were specifically targeted and lysed by AdCAR-T cells, as Figure 3B illustrates. The GFP signal started to diffuse before it disappeared completely.

Furthermore, the efficiency of target cell lysis was determined by luciferase-based cytotoxicity assay. Luciferase activity of remaining viable cells was measured, and values were normalized to untreated control organoids (lacking AdCAR-T cells and mAbs; Figure 3C). With increasing E:T ratios (0.2:1 to 5:1), the antigen-specific AdCAR-mediated cytolysis of MCF-7 organoids reached from 7% to 62% after 24 h of treatment. Thus, antigen-specific cell lysis correlated positively with the E:T ratio. The unspecific cytolysis by AdCAR-T cells (without mAbs) was relatively low (3–8%) and correlated with the E:T ratio as well. After 72 h of treatment, organoids were completely lysed, even at a low E:T of 0.6:1. No significant difference in target cell lysis was observed when comparing direct CD276 CAR T-cells on MCF-7 organoids (Figure S2).

**Figure 4:**
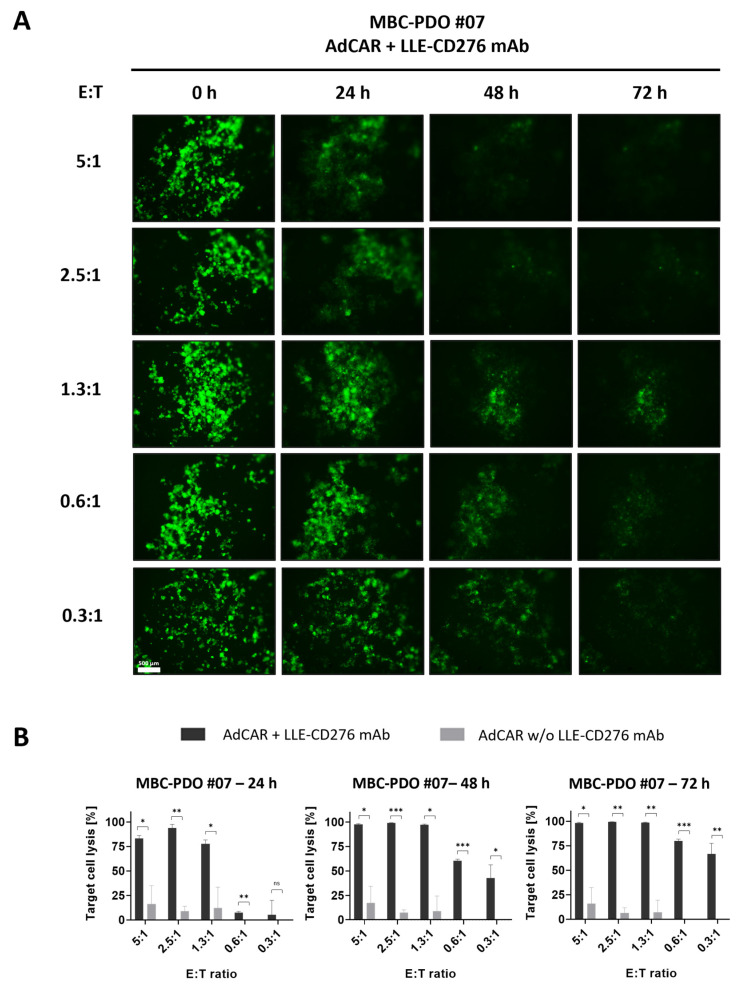
AdCAR treatment of luciferase- and GFP-expressing MBC-PDO #07 at various E:T ratios. (**A**) Fluorescence images of GFP-expressing MBC-PDO #07 treated with AdCAR-T cells of different E:T ratios with the addition of LLE-CD276 mAb over 72 h. Scale bar: 500 µm. (**B**) Target cell lysis of MBC-PDO #07 treated with AdCAR-T cells with (black bars) and without (gray bars) LLE-CD276 mAb over 72 h. Target cell lysis efficiency was determined by luciferase activity of viable organoids. Data shown represent the mean ± SD of biological triplicates (*n* = 3). Negative values were set to 0. Statistical analysis was performed using paired *t*-test. ns, not significant. * = *p* ≤ 0.05; ** = *p* ≤ 0.01; *** = *p* ≤ 0.001.

To further assess the suitability of MBC-PDOs for evaluating the efficacy of AdCAR-T, CD276-positive MBC-PDO #07 was treated with AdCAR-T cells in combination with LLE-CD276 mAb (Figure 4A,B) at different E:T ratios. Fluorescence images of GFP-expressing organoids illustrate that the GFP signal decreased and diffused with higher E:T ratios and over time (Figure 4A). Quantification of target cell lysis of MBC-PDO #07 confirmed the visual results (Figure 4B). While the addition of LLE-CD276 mAb to AdCAR-T cells led to very high target-specific lysis (up to 99%) (normalized to untreated organoids), unspecific cell lysis by AdCAR-T cells (without LLE-CD276 mAb) was relatively low (0–17%).

In summary, both MCF-7 and MBC-PDO #07 displayed high levels of CD276 antigen expression and revealed strong responses to the specific targeting by AdCAR-T cells combined with LLE-CD276 mAb. These findings suggest that our organoid lines are suitable for AdCAR treatments with various mAbs. Furthermore, the E:T ratio of 1.3:1 led to a target cell lysis of 99% after 72 h of treatment. Subsequent experiments were performed with an E:T ratio of 1:1.

### 3.4. AdCAR Treatment of MBC-PDOs Using Multiple LLE-mAbs

**Figure 5:**
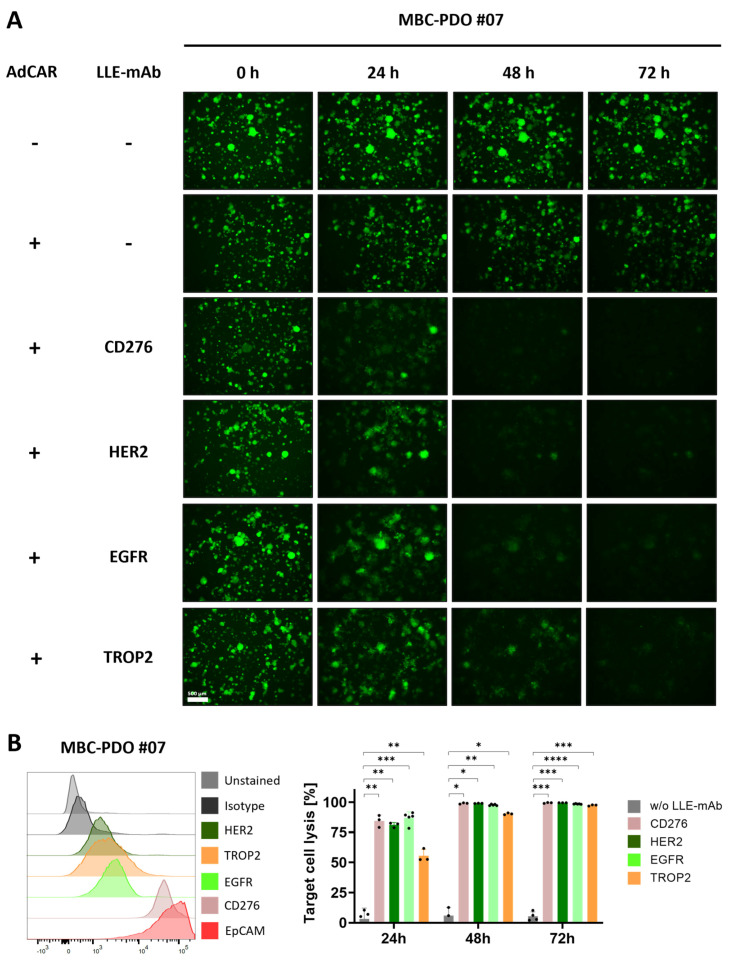
AdCAR treatment of MBC-PDO #07 with various LLE-mAbs. (**A**) Fluorescence images of GFP-expressing MBC-PDO #07 treated with AdCAR-T cells (E:T ratio was set to 1:1) with the addition of LLE-mAbs against CD276, HER2, EGFR, and TROP2. Images were taken after 0 h, 24 h, 48 h, and 72 h of treatment. Scale bar: 500 µm. (**B**) FC analysis and target cell lysis of MBC-PDO #07 treated with AdCAR-T cells (E:T ratio was set to 1:1) with (colored bars) and without (gray bars) LLE-mAbs over 72 h. Target cell lysis efficiency was determined by luciferase activity of viable organoids. Data shown represent the mean ± SD of biological triplicates (*n* = 3). Statistical analysis was performed using paired *t*-test. ns, not significant. * = *p* ≤ 0.05; ** = *p* ≤ 0.01; *** = *p* ≤ 0.001; **** = *p* ≤ 0.0001.

To investigate the potential of targeting alternative antigens beyond CD276, we tested if further LLE-mAbs have similar effects on MBC-PDO #07. Hence, this line was treated with AdCAR-T cells in combination with LLE-CD276, LLE-HER2, LLE-EGFR, and LLE-TROP2 at an E:T ratio of 1:1 (Figure 5A). Compared to the control conditions (without LLE-mAbs, +/− AdCAR), in which organoids were fully intact, treated organoids lost GFP intensity and dissolved over time. These results are in accord with the antigen expression pattern analyzed by FC, and the quantification of target cell lysis determined by luciferase activity (Figure 5B). After 24 h of treatment, LLE-CD276 and LLE-EGFR resulted in the highest lysis rates, which correlated with the antigen expression according to the FC data. At 48 h, the antigen-specific AdCAR-mediated cytolysis of MBC-PDO #07 reached from 90% to 99%, and further increased up to 100% (LLE-HER2) after 72 h of treatment.

**Figure 6:**
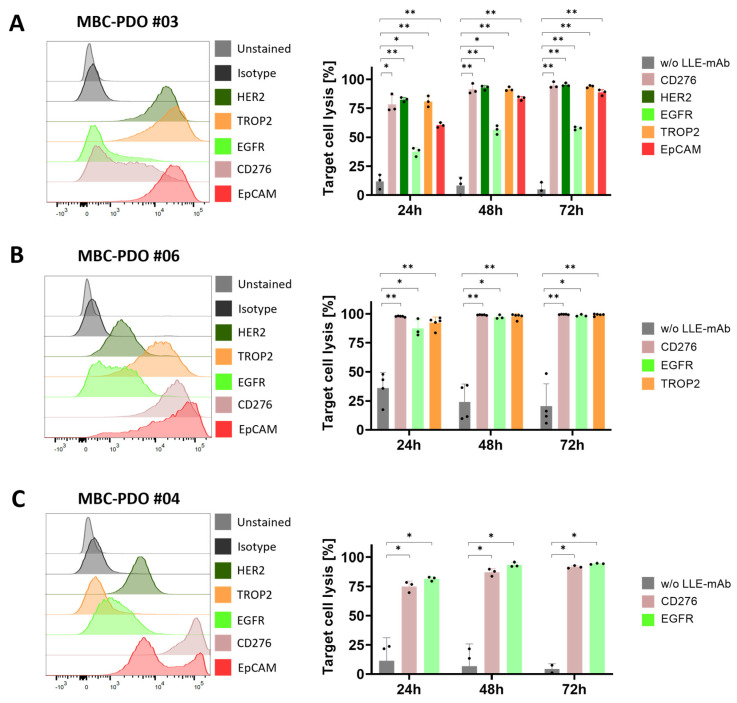
FC analysis and AdCAR treatment of MBC-PDOs with various LLE-mAbs. Treatments were performed on (**A**) MBC-PDO #03, (**B**) MBC-PDO #06, and (**C**) MBC-PDO #04. AdCAR treatment of MBC-PDOs with (colored bars) and without (gray bars) LLE-mAbs was carried out in an E:T ratio of 1:1 for 72 h. Target cell lysis efficiency was determined by luciferase activity of viable organoids. Data shown represent the mean ± SD of multiple biological replicates (*n* ≥ 3). Statistical analysis was performed using paired *t*-test. ns, not significant. * = *p* ≤ 0.05; ** = *p* ≤ 0.01.

Next, we tested the influence of AdCAR treatment and various LLE-mAbs on additional MBC-PDO lines. MBC-PDO #03 was treated with the mAbs LLE-CD276, LLE-HER2, LLE-EGFR, LLE-TROP2, and LLE-EpCAM for 72 h (Figure 6A). According to the FC data, levels of EGFR in MBC-PDO #03 were lower compared to the other antigens. Consequently, the LLE-EGFR-mediated target cell lysis was lower compared to the rest of the mAbs which achieved a lysis of up to 95%. The AdCAR treatment of MBC-PDO #06, which expressed CD276, EGFR, and TROP2, was combined with LLE-CD276, LLE-EGFR, and LLE-TROP2, respectively (Figure 6B). As expected, all three conditions led to complete cytolysis. Finally, CD276- and EGFR-expressing MBC-PDO #04 were treated with AdCAR-T cells together with LLE-CD276 and LLE-EGFR, respectively (Figure 6C). After 48 h and 72 h of treatment, the antigen-specific cytolysis achieved approx. 90%.

In conclusion, our findings illustrate that MBC-PDOs are suitable models for the in vitro screening of metastatic BC treatment by the AdCAR system and that antigen expression of MBC-PDOs correlates with their specific cytolysis mediated by AdCAR-T cells and compatible LLE-mAbs. Furthermore, the capability of targeting various antigens varied, depending on antigen expression levels and the characteristics of the target antigens that had an influence on their effectiveness in recruiting AdCAR-T cells to malignant cells. Hence, even though there is potential for universal targeting using a single CAR construct for all antigens, not all antigens were capable of recruiting AdCAR-T cells to cancer cells equally. Nevertheless, our data clearly demonstrate the potential of AdCAR-T for personalized therapies based on individual target antigen expression patterns. 

## 4. Discussion

Metastatic BC is associated with various challenges including cancer heterogeneity and treatment-resistant cells [4]. As metastatic biopsies are relatively rare, most therapy decisions depend on the characteristics of primary tumors. However, primary tumors may not represent the heterogeneous features of metastatic tumors. Therefore, therapy decisions based solely on the characteristics of primary tumors can lead to a poor outcome [6,7].

BC exhibits significant heterogeneity and can be clinically categorized into various subtypes based on the presence or absence of hormone receptors (HRs) and the status of HER2 [3]. Triple-negative BC, for one, which is defined by the absence of both HRs and HER2, is associated with a poorer prognosis, as endocrine therapy and HER2-targeted therapy are off the table [3]. In this case, alternative target antigens, expressed on the tumor cell surface, need to be explored and clinically evaluated. For instance, CD276 has shown to be expressed in numerous solid tumors including BC [26,27]. In the present study, we provide a comprehensive analysis of target antigen expression patterns in BC cell lines and MBC-PDOs, demonstrating inter-individual heterogeneity. These results clearly demonstrate the need for patient-individualized treatment approaches. 

The application of genetically engineered T cell treatments, which integrate a chimeric antigen receptor (CAR), has demonstrated impressive clinical responses in patients with hematological malignancies [18]. Thus far, multiple obstacles have restricted successful translation of CAR-T therapies in solid tumors. Guiding CAR-T cells to reach and infiltrate the tumor poses a significant challenge, which is furthermore magnified by the immunosuppressive conditions found in the tumor microenvironment [18]. Additionally, tumor cells have the capability to reduce antigen expression under the selective pressure exerted by CAR-T cells. Promiscuous antigen expression between tumor and physiological tissues can result in on-target off-tumor effects and associated life-threatening toxicities [18]. This is particularly highlighted in the case study conducted by Morgan et al., where the application of HER2-targeted CAR-T cells triggered a cascading systemic inflammation, ultimately resulting in multi-organ failure that was attributed to CAR-T activation by a low-level expression of HER2 on lung epithelial cells [28]. This emphasizes the necessity for advancements in the emerging field of CAR-T cell-based therapy in BC, where progress depends on discovering suitable TAAs and mechanisms for stringent control of CAR-T activity. This is especially pronounced in the context of triple-negative BC.

To date, numerous novel CAR-based target antigens have been evaluated against BC. The majority of these studies have been conducted in preclinical trials. Ongoing clinical trials with BC patients include the targeting of HER2, GD2, and EpCAM amongst others [19]. In addition to targeting tumor cells, efforts have been directed toward the elimination of cells residing within the extracellular matrix [29]. Combinatorial strategies with CAR-T cells and conventional BC therapy may lead to better efficacy, especially in terms of overcoming the suppressive tumor microenvironment. These approaches have yet to be assessed in clinical trials. 

One elegant way to improve safety and flexibility of CAR-T cells in comparison to conventional CAR-T cell designs is to split antigen recognition from CAR-T cell activation. By introducing “adapter” molecules that bridge between TAA and CAR, this approach allows maximal control of CAR-T cell activity. Pioneered by the expression of an Fcγ receptor (CD16) [30] or CD16-derived CAR construct [31] in T cells to enable antibody-dependent cellular cytotoxicity (ADCC), multiple groups have re-invented the concept of “adapter”-mediated CAR-T activation [32,33,34,35]. We have recently reported on the development of the AdCAR platform [23]. The AdCAR is directed against biotin in the context of a specific linker structure, referred to as linker–label–epitope (LLE). We did not see any interference with serum or protein-bound biotin. The LLE-tag can be chemically conjugated on any kind of binding molecule (e.g., mAbs, mAb fragments, natural or synthetic ligands), allowing highly flexible and convenient AM generation. This flexibility in AM generation provides an advantage over AMs that incorporate recombinant tags [32,33,35], facilitating to build on clinically available mAbs. Moreover, we use the physiologically available vitamin biotin as a label. In contrast to other approaches using, for example, FITC, we expect less immunogenicity. Inherent to the design of all “adapter”-CAR systems is the beneficial safety profile, rendering these approaches a perfect fit for promiscuously expressed TAA. Moreover, AdCAR-T allows highly flexible and multiple targeting to prevent antigen escape and enable individualized targeting the regiment’s platform [23]. First “adapter”-CAR systems have already entered the clinic (NCT04230265), targeting CD123 in adult AML, demonstrating complete remission in 2 out of 3 patients and underscoring the feasibility of “adapter”-CAR approaches [36]. To explore this concept, we tested AdCAR-T cells with a variety of adapter molecules that target different antigens on MCF-7 and triple-negative MDA-MB-468 in 2D cultures (Figure 1C–F). Most of the targets used in this study harbor clinical relevance [19]. We observed a positive correlation between antigen expression and target cell lysis.

To demonstrate the feasibility of patient-individualized targeting and co-clinical functional validation, we utilized our MBC-PDOs’ platform [14]. As shown before, MBC-PDOs preserve both receptor statuses and hotspot mutations across numerous passages. Therefore, MBC-PDOs provide a reliable in vitro platform for pre-clinical assessment of therapeutic effectiveness [14]. For the first time, we applied this platform for systematic testing in the context of immunotherapy, particularly CAR-T cells. In accordance with individual antigen expression profiles assessed by flow cytometry (Figure 2B), AdCAR-T in combination with specific adapter molecules delivered potent lysis of MBC-PDOs. Specific lyses strongly correlated with antigen expression. For instance, MBC-PDO #03 demonstrated minor levels of EGFR and high levels of CD276, HER2, TROP2, and EpCAM expression (Figure 6A). As a result, AdCAR-mediated cytolysis applying LLE-EGFR mAbs was low (38–58%), while the treatments with LLE-CD276, LLE-HER2, LLE-TROP2, and LLE-EpCAM led to complete target cell lysis. Future studies can include combinatorial approaches with multiple targets, as this was demonstrated to be superior compared to monotargeting [24]. Whether targeting specific TAAs results in enhanced target cell lysis can be investigated in further studies While our approach is clearly functional and shows a correlation between antigen expression and target cell lysis in 2D culture (Figure 1E,F), further investigation is needed within the 3D setting. Here, antigen staining alone does not seem to provide a clear prediction of AdCAR activity. 

We, however, clearly demonstrate the feasibility of individually analyzing target antigen expression profiles and functionally validating patient-derived organoids’ efficacy of specific targeting. We further underscore the potential of AdCAR-T as an ideal tool for precision immunotherapy allowing individual selection target antigens, streamlining complex manufacturing and safety assessment of engineered cellular therapeutics. By building clinically tested and approved antibodies, this approach will facilitate clinical translation and accessibility of targeted cellular therapies to a very heterogeneous patient cohort. 

Our MBC-PDO-based in vitro platform in combination with AdCAR-T cells pave the way for precision immunotherapy in solid tumor malignancies and can further be utilized in the assessment of existing and novel therapeutic approaches.

## 5. Conclusions

In conclusion, MBC-PDO lines serve as a reliable in vitro platform that can be utilized in the assessment of AdCAR-T cell effectiveness in treating metastatic BC. Further research is required to demonstrate the soundness of these promising models for clinical relevance. As a minuscule number of BC patients develop MA or MPE, further sources for metastatic biopsies should be introduced as a novel standard in pathological and experimental applications. Consequently, the biobank of patient-derived BC organoid models can be extended to increase access to this pre-clinical evaluation platform. This study clearly demonstrates the feasibility of precision immunotherapy utilizing AdCAR-T to target patient-individualized antigen patterns.

## Data Availability

The data demonstrated in this study are available upon request from the corresponding author.

## Supplementary Materials

The following supporting information can be downloaded at: https://www.mdpi.com/article/10.3390/cancers16010168/s1, Table S1: List of BC patient data from whom the organoid lines were established. Table S2: Composition of breast cancer medium (BCM). Table S3: Antibody information chart. Figure S1: Correlation between LCA and real-time ICA. Figure S2: Direct CD276 CAR-T cell treatment of MCF-7 organoids expressing GFP.
