# Supplementary material for: Precision Immunotherapy Utilizing Adapter CAR-T Cells (AdCAR-T) in Metastatic Breast Cancer Leads to Target Specific Lysis
Source: Cancers (Basel). 2023 Dec 29;16(1):168. doi: 10.3390/cancers16010168 (PMC10778501; doi:10.3390/cancers16010168)
Supplement: Supplementary file 1 [file cancers-16-00168-s001.zip › cancers-2720715-supplementary.pdf]

cancers MDPI

Precision Immunotherapy Utilizing Adapter CAR-T Cells (AdCAR-T) in Metastatic Breast Cancer Leads to Target Specific Lysis

Cansu E. Önder, Moustafa Moustafa-Oglou, Sarah M. Schröder, Andreas D. Hartkopf, André Koch, Christian M. Seitz

Table S1. List of BC patient data from whom the organoid lines were established. All patients were female aged between 52 and 70. Abbreviation: NST (no special type).

MBC-PDO	#03	#04	#06	#07
**Primary tumor**	NST (2008)	NST (2009)	NST (2019)	NST (2019)
**Therapy of primary tumor**	Trastuzumab; Aromasin; Carboplatin; Taxol; Avastin (until 2019)	FEC + Docetaxel; Tamoxifen (until 2013)	Epirubicin/Cyclophosphamide (aborted)	Doxorubicin
**Metastasis**	Hepatic and pleural metastasis (2019)	Lymph nodes (2017) Hepatic metastasis (2021)	Hepatic and osseus metastasis (2020) Pulmonary and medullary metastasis (2021)	Cutaneous, lymphogenic and osseus metastasis (2020-2021)
**Therapy of Metastasis**	Avastin	Ribociclib + Letrozole; Palbociclib + Letrozole	Letrozole + Goserelin + Abemaciclib	Paclitaxel; Eribulin; Abemaciclib + Letrozole
**Therapy prior to drainage**	Avastin; Nab-Paclitaxel; Eribulin (2020)	Abemaciclib + Fulvestrant (2020-2021)	Paclitaxel (2021)	Capecitabin (2021)
**Drainage**	Pleura (2021)	Pleura (2021)	Pleura (2021)	Pleura (2021)
**Follow-up therapy**	Palbociclib + Letrozole; Vinorelbine + Trastuzumab + Pertuzumab	Everolimus + Aromasin; Doxorubicin	None, patient did not survive	Carboplatin + Olaparib; Olaparib
**Additional information**	Hepatic and pleural metastasis (2019): NST infiltration, *PIK3CA* H1047R mutation	-	-	NST (2019): *BRCA1/2* deletion, *AKT1* E17K mutation

Table S2. Composition of Breast Cancer Medium (BCM). Composition was previously described [24, 27].

Component	Final concentration	Company	Catalog number
L-WRN conditioned medium	50%	Home-made	-
Neuregulin 1	5 nM	Peprotech (Cranbury, NJ, USA)	100-03
FGF7	5 ng/mL	Peprotech (Cranbury, NJ, USA)	100-19
FGF10	20 ng/mL	Peprotech (Cranbury, NJ, USA)	100-26
EGF	5 ng/mL	Peprotech (Cranbury, NJ, USA)	AF-100-15
A83-01	500 nM	Tocris Bioscience (Bristol, UK)	2939
Y-27632	5 µM	Hölzel Diagnostika Handels GmbH (Köln, Germany)	TMO-T1725-50 mg
SB202190	500 nM	Sigma-Aldrich (St. Louis, MO, USA)	S7067
B-27™ supplement	1x	Thermo Fisher Scientific (Waltham, MA, USA)	17504-44
Nicotinamide (NIC)	5 mM	Sigma-Aldrich (St. Louis, MO, USA)	NO636
N-Acetylcysteine (NAC)	1.25 mM	Sigma-Aldrich (St. Louis, MO, USA)	A9165-5G
Primocin	50 µg/mL	InvivoGen (San Diego, CA, USA)	Ant-pm-1
AdvDMEM +++ (1% Pen/Strep, 1x GlutaMAX™-I, 10 mM HEPES)	1x	Thermo Fisher Scientific (Waltham, MA, USA)	12634-028; 15140-122; 35050-038; 15630-056

Table S3. Antibody information chart.

Antibody	Clone	Host	Isotype	Conjugation	Company	Cat No.
Anti-CD47	REA220	human Hybridoma cell line	recombinant human IgG1	Biotin	Miltenyi Biotec	130-101-342
anti-CD66	TET2	human Hybridoma cell line	recombinant human IgG1	Biotin	Miltenyi Biotec	130-093-156
anti-CD112	R2.525	human Hybridoma cell line	recombinant human IgG1	Biotin	Miltenyi Biotec	130-109-000
anti-CD133	REA820	human Hybridoma cell line	recombinant human IgG1	Biotin	Miltenyi Biotec	130-112-193
anti-CD146	541-10B2	human Hybridoma cell line	recombinant human IgG1	Biotin	Miltenyi Biotec	130-092-850
anti-CD171	REA163	human Hybridoma cell line	recombinant human IgG1	Biotin	Miltenyi Biotec	130-100-702
anti-CD276	REA1094	human Hybridoma cell line	recombinant human IgG1	Biotin	Miltenyi Biotec	130-118-579
anti-ROR1	REA1051	human Hybridoma cell line	recombinant human IgG1	Biotin	Miltenyi Biotec	130-118-018
anti-CD326	REA764	human Hybridoma cell line	recombinant human IgG1	Biotin	Miltenyi Biotec	130-111-114
anti-SSEA	REA101	human Hybridoma cell line	recombinant human IgG1	Biotin	Miltenyi Biotec	130-098-339
anti-TROP2	REA916	human Hybridoma cell line	recombinant human IgG1	Biotin	Miltenyi Biotec	130-115-096
anti-Biotin	REA746	human Hybridoma cell line	recombinant human IgG1	APC	Miltenyi Biotec	130-111-069
anti-HER2	Trastuzumab, Kanjinti	Chinese Hamster Ovarian	IgG1 Isotype	Biotin	Pharmacy University clinics Tübingen UKT	1144554A
anti-EGFR	Cetixumab, Erbitux	murine myeloma cell line	IgG1 Isotype	Biotin	Pharmacy University clinics Tübingen UKT	G0157D

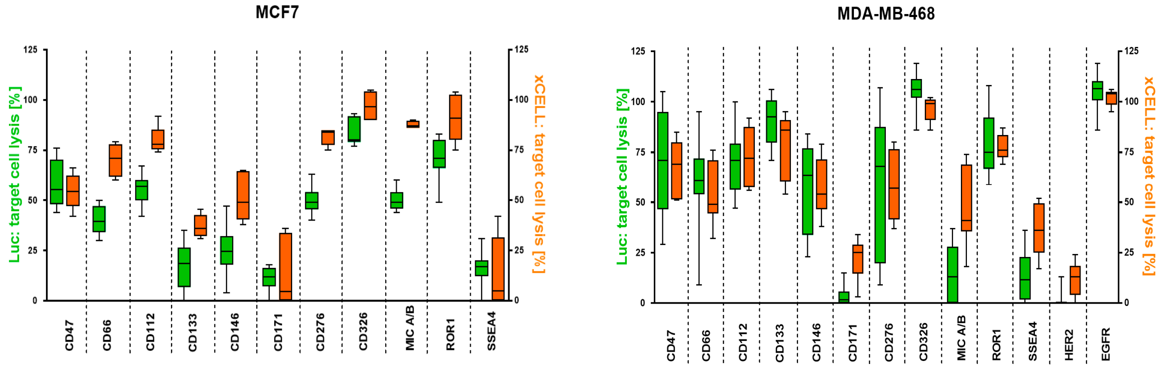

Figure S1. Correlation between LCA and real-time ICA. Comparison of target cell lysis of MCF-7 and MB-MDA-468 via LCA (green bars) or ICA (red bars). Target cell lysis with an E:T ratio of 2:1 via LCA and ICA was determined after 48h and 24h respectively. Data is represented by mean values (n=6) ± SD.

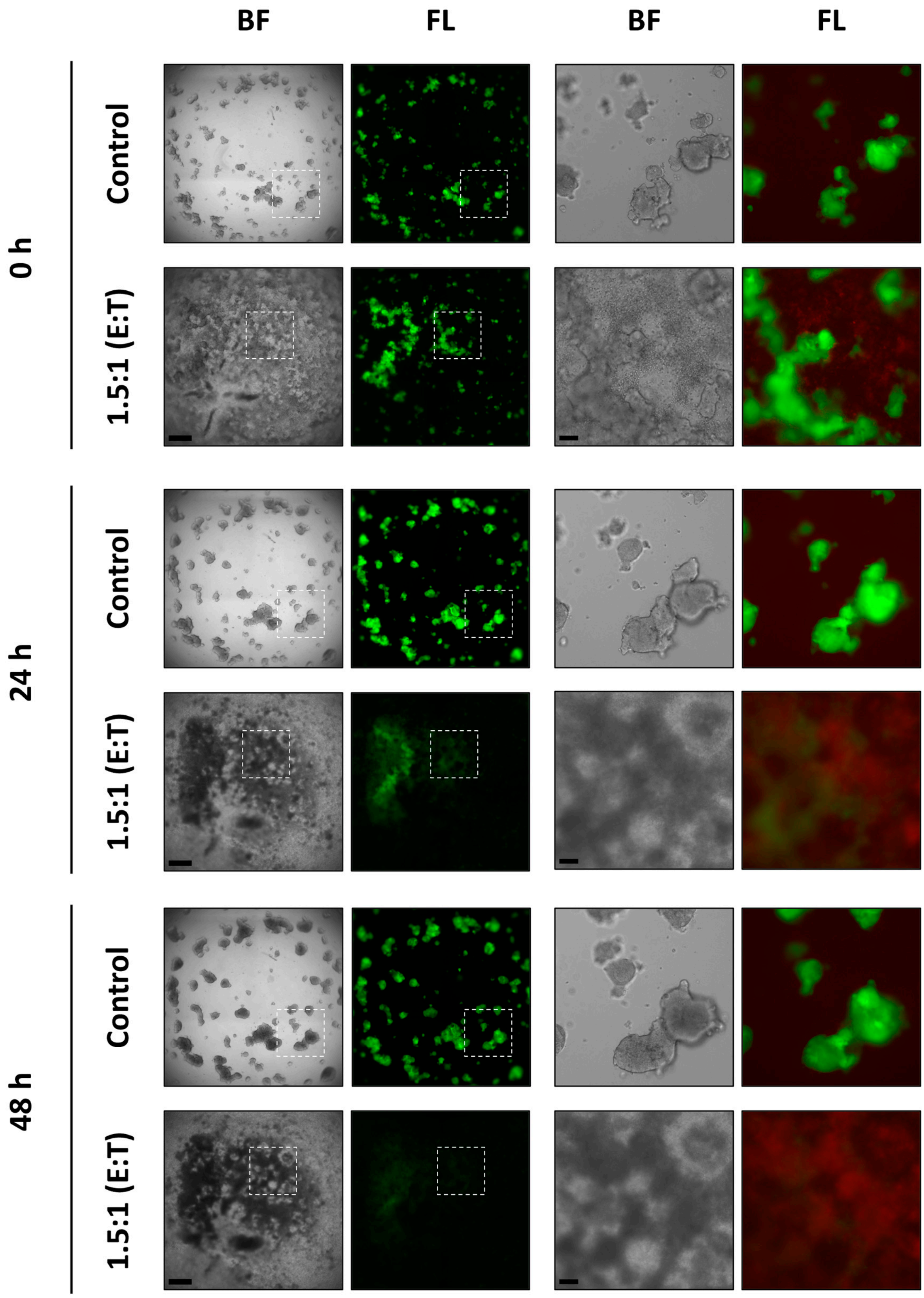

Figure S2. Direct CD276 CAR-T cell treatment of MCF-7 organoids expressing GFP. Brightfield (BF) and fluorescence (FL) images of GFP-expressing MCF-7 organoids (green) treated with mCherry-expressing CD276 CAR-T cells (red) in an E:T ratio of 1.5:1 for 48 h. Control conditions represent MCF-7 organoids alone. Scale bar of left two columns: 500 µm. Scale bar of right two columns: 100 µm.

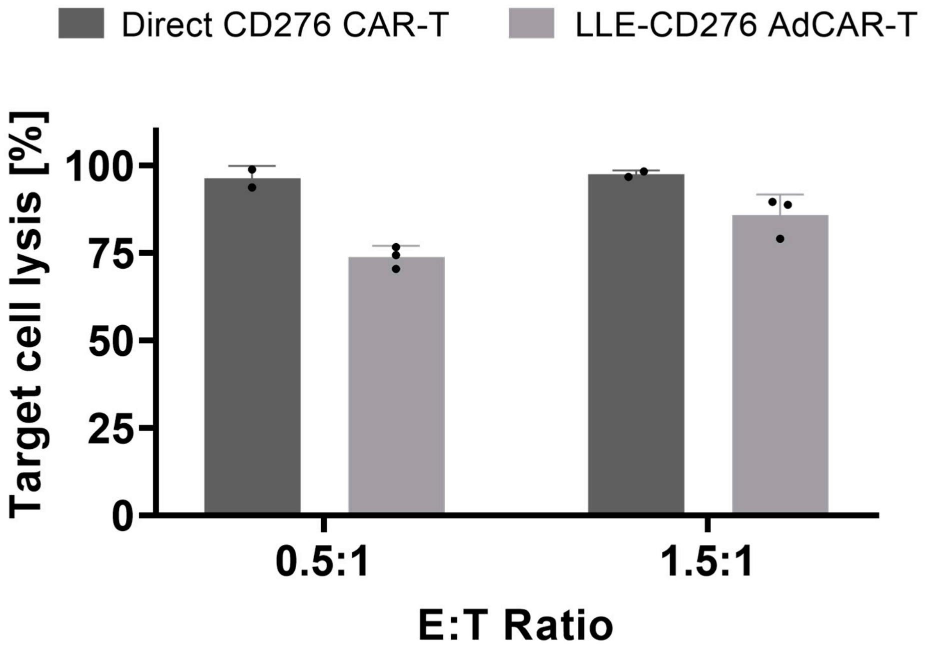

Figure S3. Comparison of target cell lysis between direct CD276 CAR-T and LLE-CD276 AdCAR-T on MCF-7 organoids at an E:T ratio of 0.5:1 and 1.5:1 after 48h. Target cell lysis was normalized to MCF-7 organoids alone. Data shown represents the mean ± SD of biological duplicates (n = 2) and triplicates (n = 3) for direct CD276 CAR-T and LLE-CD276 AdCAR-T respectively.

Disclaimer/Publisher's Note: The statements, opinions and data contained in all publications are solely those of the individual author(s) and contributor(s) and not of MDPI and/or the editor(s). MDPI and/or the editor(s) disclaim responsibility for any injury to people or property resulting from any ideas, methods, instructions or products referred to in the content.
